# Paraneoplastic Dermatoses: A Clue for Underlying Malignancies

**DOI:** 10.3390/jcm14031014

**Published:** 2025-02-05

**Authors:** Dario Didona, Alessandra Rallo, Andrea Carugno, Giovanni Paolino

**Affiliations:** 1 Rare Diseases Unit, Istituto Dermopatico dell’Immacolata (IDI)-IRCCS, 00167 Rome, Italy; d.didona@idi.it (D.D.); a.rallo@idi.it (A.R.); 2 Dermatology Unit, Department of Clinical Internal, Anesthesiological and Cardiovascular Science, University of La Sapienza, 00185 Rome, Italy; 3 Dermatology Unit, Department of Medicine and Surgery, University of Insubria, 21100 Varese, Italy; 4 Unit of Dermatology, IRCCS Ospedale San Raffaele, 20132 Milan, Italy; paolino.giovanni@hsr.it

**Keywords:** acanthosis nigricans, dermatomyositis, Leser–Trélat’s sign, neoplasia, paraneoplastic dermatoses, paraneoplastic autoimmune multiorgan syndrome, pyoderma gangrenosum, Sweet’s syndrome, tripe palm

## Abstract

Paraneoplastic dermatoses (PDs) belong to a group of rare and polymorphous dermatoses, and they can often be the first sign of underlying malignancies. Therefore, dermatologists should be able to identify skin features to identify earlier underlying neoplasms. Indeed, lack of familiarity with cutaneous clues of internal malignancies can lead to a delay in the diagnosis and an impairment of the prognosis of the patients. In this review, we described several PDs, including more common and rarer PD. Indeed, while malignant acanthosis nigricans, characterized by velvety, verrucous, hyperpigmented plaques that usually affect intertriginous areas, is a well-known PD, necrolytic migratory erythema is usually misdiagnosed because its clinical features can be similar to seborrheic dermatitis. Furthermore, we focused on two paradigmatic PDs, namely paraneoplastic autoimmune multiorgan syndrome (PAMS) and paraneoplastic dermatomyositis. Indeed, PAMS represents a paradigmatic form of obligate PD, which is always associated with an underlying neoplasm, while paraneoplastic dermatomyositis belongs to the facultative PD, which can be associated with neoplasia in a variable percentage of cases.

## 1. Introduction

Paraneoplastic dermatoses (PDs) are a heterogeneous group of rare skin diseases characterized by the presence of an underlying neoplasia. In 1868, the famous dermatologist von Hebra proposed the idea that sudden alterations of cutaneous pigmentation could be associated with an occult neoplasia [1,2,3]. Several years later, Curth proposed six criteria to identify a PD (Table 1) [1,2,3].

The pathogenesis of PD is still unclear and depends on the underlying neoplasia, which can manifest before, after or even simultaneously in relation to the development of PD [1,2,3]. PD can be classified into obligate PD and facultative PD, depending on the rate of the association between the skin manifestation and the underlying neoplasia. Indeed, obligate PDs are always associated with an underlying malignancy, while facultative PDs are statistically associated with neoplasia, but also with other non-neoplastic diseases, like inflammatory bowel diseases (IBDs) [1,2,3]. Since several PD have been reported in the literature (Table 2) and a complete list of them is beyond the purposes of our review, we focused on necrolytic migratory erythema (NME), paraneoplastic autoimmune multiorgan syndrome (PAMS) and tripe palm (TP) for the group of obligate PD, and on acanthosis nigricans (AN), Leser–Trélat’s sign (LTS), paraneoplastic dermatomyositis (DM), pyoderma gangrenosum (PG) and Sweet’s syndrome (SS) for the group of facultative PDs.

## 2. Obligate Paraneoplastic Dermatoses

### 2.1. Necrolytic Migratory Erythema

**Figure 1:**
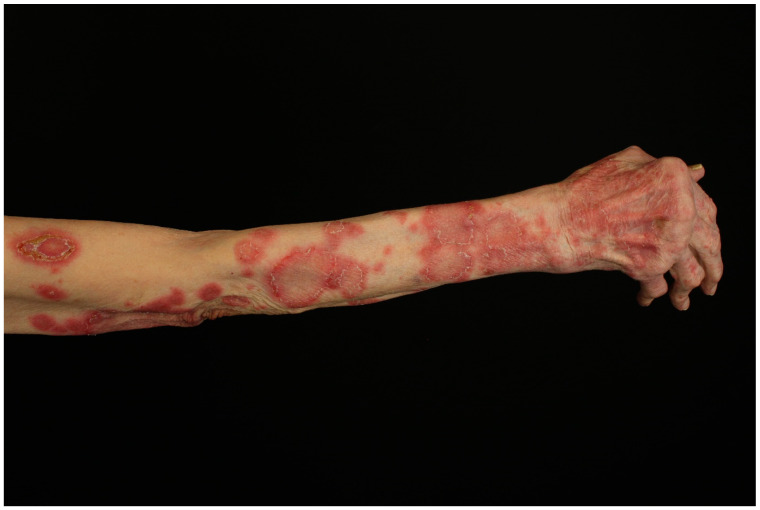
Necrolytic migratory erythema in a female patient with glucagonoma.

Necrolytic migratory erythema (NME) is a paraneoplastic skin manifestation associated with an underlying glucagonoma, a rare pancreatic tumor composed by pancreatic alpha cells that secrete an excessive amount of glucagon [4,5]. Glucagonoma is extremely rare, and its incidence is estimated between 0.01 and 0.1 new cases per 100,000 inhabitants per year [6]. NME represents a manifestation of glucagonoma syndrome, which includes also angular cheilitis, glossitis, diabetes mellitus type 2, normochromic anemia, weight loss, venous thrombosis, neurological and neuropsychiatric disorders, like depression, psychosis, ataxia, and proximal muscle weakness [4,5]. The association between skin manifestations and pancreatic neoplasia was first described by Becker et al. in a patient with an alpha-cell tumor of the pancreas [7] and in 1973, the term NME was introduced by Wilkinson to describe a characteristic rash, characterized by irregular, often partly circinate, erythematous eruptions with pseudo-bulla formation [8]. The pathogenesis of NME is still not clear. On the one hand, a pivotal role of hyperglucagonemia has been proposed, because surgical removal of the tumor and medical control of glucagon levels often lead to the resolution of the clinical features; on the other hand, hyperglucagonemia alone does not fully explain the development of NME [9,10]. Indeed, NME can also occur in pseudo-glucagonoma syndrome, characterized by a lack of elevated glucagon levels and pancreatic neoplasia [11,12]. In addition, liver dysfunction, with decreased glucagon clearance and low albumin levels, may contribute to NME development by exacerbating nutrient deficiencies [13]. NME typically presents as pruritic, painful erythematous vesicles and blisters that can rapidly evolve into crusted, ulcerated plaques with irregular borders (Figure 1) [4,5].

NME can be widespread or localized and typically involves the perioral area, trunk, groin, intergluteal fold, genitals, and acral areas [4,5]. The management of glucagonoma relies on surgical resection of the tumor, which can lead to complete resolution of both cutaneous and systemic symptoms [4]. Medical therapies include chemotherapy, somatostatin analogs, and targeted molecular therapies, like sunitinib and everolimus [14]. Several differential diagnoses should be ruled out, including acrodermatitis enteropathica, pemphigus foliaceus (PF), seborrheic dermatitis. Acrodermatitis enteropathica is an autosomal recessive disorder due to a variation in the intestinal zinc transporter gene *SLC39A4* and it is characterized by a triad of perioral, flexural, and acral dermatitis, alopecia, and diarrhea [15]. Therefore, the evaluation of serum zinc levels is extremely helpful to differentiate acrodermatitis enteropathica from NME. PF, a rare autoimmune blistering disease, can be easily ruled out by performing direct immunofluorescence (DIF) microscopy, indirect immunofluorescence (IIF) microscopy and serological examinations, which are always negative in NME [16]. Finally, seborrheic dermatitis is an extremely common dermatitis that involves typically seborrheic skin areas and more frequently the face [17]. It is characterized by fine scales on erythematous skin and can be easily distinguished from NME because of its clinical distribution and responsiveness to topical antifungal and corticosteroid therapy [17]. 

### 2.2. Paraneoplastic Autoimmune Multiorgan Syndrome

**Figure 2:**
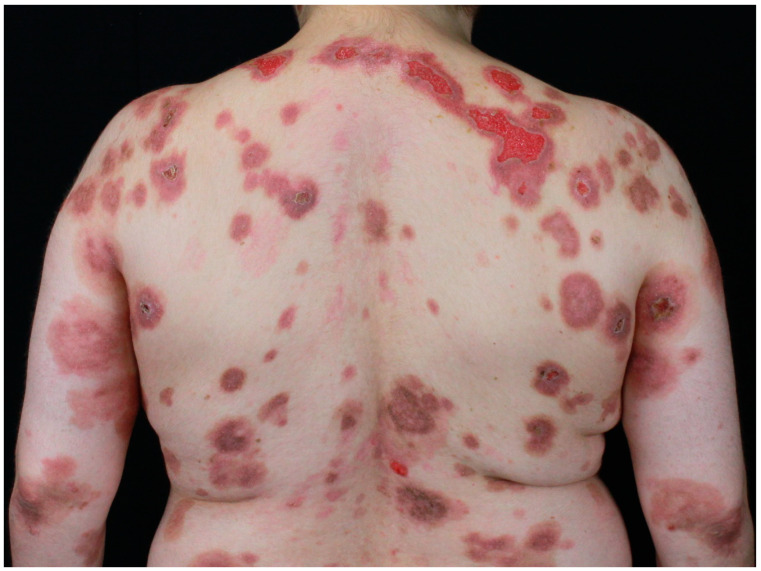
Skin erosions and post-inflammatory macules in a female patient with paraneoplastic autoimmune multiorgan syndrome and non-Hodgkin lymphoma.

PAMS, previously known as paraneoplastic pemphigus (PNP), belongs to the group of obligate PD [1]. In 1990, PNP was formally described as a specific entity [18], although the association between pemphigus and neoplasms has been previously reported [19]. The term PAMS was proposed in 2001 to better highlight the different profile from that of patients with pemphigus vulgaris (PV) and underlying neoplasia, which is characterized by polymorphous mucocutaneous lesions, immunologic abnormalities, and potential multiorgan involvement [20]. PAMS belongs to the group of pemphigus diseases, which also includes PV, pemphigus foliaceus and IgA pemphigus, characterized by autoantibodies directed against cell surface molecules of keratinocytes, resulting in a loss of keratinocyte adhesion [16]. In up to 50% of cases, the presentation of PAMS precedes the diagnosis of an underlying neoplasm, which mostly belongs to lymphoproliferative disorders [21]. Indeed, it has been pointed out that non-Hodgkin lymphoma is the most associated neoplasia with PAMS, followed by chronic lymphocytic leukemia and Castleman’s disease [22]. Moreover, thymoma, Waldenstrom’s macroglobulinemia, Hodgkin’s lymphoma, and monoclonal gammopathy have also been reported in association with PAMS [23,24]. Furthermore, adenocarcinomas and squamous cell carcinomas have been reported in 8.4% of cases, while sarcomas have been detected in 6.2% of cases [23,24]. In addition, several PAMS cases have been reported as iatrogenic, although it is not clear whether these cases were truly iatrogenic or whether the association with chemotherapy or radiation was merely coincidental [25,26,27,28,29,30,31]. Interestingly, all these reported cases were associated with an underlying lymphoproliferative malignancy. The pathogenesis of PAMS has not been completely elucidated. An association with the HLA-DRB1*03 allele in French Caucasian patients [32] as well as with the HLA-Cw*14 allele in Chinese patients has been reported [33]. Several antigenic targets have been described in PAMS patients. Plakins represent the major antigenic target and include periplakin (190 kDa), desmoplakin II (210 kDa), envoplakin (210 kDa), bullous pemphigoid antigen 1 (BP230, 230 kDa), desmoplakin I (250 kDa), epiplakin (500 kDa), and plectin (500 kDa) PMID: 30103046 PMID: 11176692. The second most important antigenic target is represented by the plakins group, which include anti-desmoglein (Dsg) 1 and 3 antibodies, as well as desmocollins [20,34]. However, the pathogenicity of anti-Dsg3 autoantibodies is still unclear. Indeed, PAMS can occur without anti-Dsg3 autoantibodies [35], but anti-Dsg3 autoantibodies induce acantholysis in vivo in neonatal mice [36]. In addition, autoantibodies against α-2 microglobulin-like 1 (A2ML1), a 170 kDa protease inhibitor expressed in the epidermis, have been detected in 50–79% of patients with PAMS [34]. Clinical features of PAMS are extremely polymorphous, and both the skin and different mucosae can be affected. The heterogeneity of the clinical picture is probably related to the different antibodies that are involved in PAMS [23,37]. Indeed, it has been found out that pemphigus-like lesions (blisters and erosions) (Figure 2) are related to humoral-mediated cytotoxicity, while a lichenoid phenotype (papules, plaques and erythema) is related to cell-mediated cytotoxicity [34]. 

In about 30% of PAMS, an underlying neoplasia can be detected after the first clinical skin or mucosal manifestation and oral erosions or ulcerations usually represent the first clinical manifestation of PAMS [1,24]. The bronchopulmonary system is affected in up to 90% of cases, usually with obstructive lung disease and bronchiolitis obliterans (BO) [34]. In addition, BO is one of the leading causes of death in PAMS [38]. Although the underlying pathogenesis of BO is still not clear, anti-epiplakin and anti-Dsg1 antibodies have been reported as related to BO development [39,40]. Other potentially affected organs include the upper and lower gastrointestinal tract, thyroid gland, kidneys, and smooth muscle tissue, although their involvement seems to be most likely due to associated diseases, such as autoimmune thyroid disease and myasthenia gravis [41,42]. The diagnosis of PAMS relies on clinical features, DIF microscopy, IIF microscopy and serological examinations [43]. DIF microscopy on perilesional skin or mucosa usually detects intercellular deposits of IgG and/or C3 in a so-called “net-like” staining pattern within the epidermis [44]. Furthermore, linear or granular deposits of IgG and/or C3 along the basal membrane zone (BMZ) may also be found [44]. IIF can be performed using different substrates. IIF on monkey esophagus usually detects intercellular deposition of IgG, showing a sensitivity between 68% and 100% [40]. However, rat bladder epithelium has been reported as the most sensitive and specific IIF substrate for PAMS diagnosis due to its high amounts of plakins [40]. Indeed, it has been reported that 86% of PAMS patients showed reactivity by IIF using a rat bladder with an almost 100% specificity [44]. Several methods can be used to detect autoantibodies in PAMS, including enzyme-linked immunosorbent assay (ELISA), immunoblotting (IB) and immunoprecipitation (IP). However, IB and IP are usually available only in specialized laboratories. In patients affected by PAMS, autoantibodies directed against different antigens can be detected, including members of the plakin family, such as envoplakin, periplakin, desmoplakin, BP230, and desmosomal cadherins, such as Dsg1, Dsg3 and desmocollins [45,46]. It has been reported that antibodies against envoplakin and periplakin can be found in up to 88% of the patients, while epiplakin, plectin and BP230 can be detected in 61%, 57% and 30%, respectively [43,47]. An ELISA for the detection of anti-envoplakin antibodies is commercially available and shows a specificity of almost 99% [46]. In addition, ELISA can detect autoantibodies against Dsg3 and Dsg1 in between 78.8% and 100% and in between 13.3% and 26% of PAMS sera, respectively [40,48]. Several different diagnostic criteria for the classification of PAMS have been proposed, but none have been universally accepted or validated so far [18,49,50]. Recently, new criteria for diagnosing PAMS have been proposed [43] (Table 3). 

Several diagnoses should be ruled out before diagnosis of PAMS, including drug eruptions, like lichenoid drug eruptions and toxic epidermal necrolysis [24]. In these cases, accurate anamnesis is of pivotal importance to differentiate between a drug-induced disease and an autoimmune disease like PAMS. The therapy of PAMS represents a challenge. An improvement of the clinical picture has been reported after treatment or removal of the underlying malignancy [43,47]. Although a golden-standard approach has been not yet identified, systemic corticosteroids in association with steroid-sparing agents (e.g., azathioprine and mycophenolate mofetil) still represent a mainstay of the therapy of PAMS [18,49,50]. Rituximab has been reported as effective in PAMS associated with B-cell malignancies [51]. Furthermore, high doses of intravenous immunoglobulins have also been used [43,47].

### 2.3. Tripe Palms

TP, also known as acquired pachydermatoglyphia, is characterized by hyperkeratotic, yellowish, velvety thickening of the normal dermatoglyphics of the hands and fingers, often accompanied by cobbling and honeycombing patterns [52]. TP is associated mostly with lung and gastrointestinal malignancies and less frequently with breast and genitourinary neoplasia [2,53]. TP typically manifests before the diagnosis of the underlying malignancy [52]. In addition, malignant AN (MAN) and LTS are usually associated with TP [3,54,55]. Indeed, it has been proposed that TP could be a localized form of MAN [56,57]. Although the pathogenesis of TP remains still largely unknown, it has been proposed that upregulation of epidermal growth factor receptor (EGFR) and fibroblast growth factor receptor 3 (FGFR3), as well as the secretion of tumor growth factor α (TGF-α), insulin growth factor 1 (IGF-1), and epidermal growth factor (EGF) by tumor cells, can play a pivotal role [57,58]. Indeed, TGF-α plays a role in keratinocyte proliferation, binding to EGFR and leading to the activation of the mitogen-activated protein kinase (MAPK) and the extracellular-signal-regulated kinase (ERK) [57,58]. Several dermatoses can resemble TP, including primary hypertrophic osteoarthropathy (HOA) and palmoplantar psoriasis. Primary HOA is a rare genetic disease, characterized by digital clubbing, periostosis, and pachydermia and two peak of incidence, namely the first year of life and during the puberty [59]. Palmoplantar psoriasis is a common subtype of psoriasis that usually shows hyperkeratotic and erythematous plaques with symmetrical distribution on the palmar and/or plantar regions, in association with painful fissures [60]. Furthermore, it is characterized by a chronic course with recurrent flares, which are responsive to several therapies, including topical corticosteroids and systemic retinoids [60].

## 3. Facultative Paraneoplastic Dermatoses

### 3.1. Acanthosis Nigricans

AN manifests with symmetric velvety, verrucous, hyperpigmented plaques, most often in intertriginous areas, such as the axilla and the neck, although any skin area can be involved [61]. AN can be a manifestation of endocrinological and metabolic diseases, including diabetes mellitus, obesity and insulin resistance [61]. Furthermore, AN can represent a facultative PD, also known as MAN. MAN is usually associated with intra-abdominal malignancies; among them, gastric adenocarcinoma represents the most common one [1,2]. Furthermore, MAN has been reported in association with mycosis fungoides (MF) [62,63,64]. Interestingly, MF can share some clinical aspects with MAN, showing vegetating or papillomatous plaques in the skin folds [65,66]. In case of an underlying malignancy, AN usually shows a sudden and widespread onset in velvety hyperkeratotic plaques, commonly surrounded by acrochordons. Generalized pruritus can also be present [1,2]. Involvement of oral, anal and genital mucosa has also been described [1,2]. In this case, MAN can represent a challenging differential diagnosis resembling papilloma [61]. MAN can be associated with TP and LTS [1,2]. Histologically, MAN is characterized by hyperkeratosis, papillomatosis, and acanthosis and does not differ from AN [3]. Hyperpigmentation of the basal layer can also be observed [3]. The underlying mechanism of developing MAN is not completely understood. In patients with solid tumors, the development of AN may be a result of a proliferative hint induced by different cytokines, like TGF-α, IGF-1, fibroblast growth factor (FGF), and melanocyte-stimulating hormone (MSH) [3,61]. More specifically, TGF-α binds to EGFR and leads to the activation of MAPK and ERK [3,61]. Furthermore, an increase in EGFR and ERK activity has been demonstrated in keratinocytes from MAN skin samples. MAN usually improves after the treatment of the underlying neoplasm, while a worsening or relapses of AN can be an important clue of resistance to treatments, progression or recurrence of the neoplasm [3]. AN should be differentiated from terra firma-forme dermatosis, which is characterized by brownish-gray macules and patches with a typical dirt-like appearance that disappears after application of isopropyl alcohol [67].

### 3.2. Leser–Trélat’s Sign

LTS is characterized by the sudden eruption of multiple seborrheic keratoses (SK). It was first described independently by Leser and Trélat in the 19th century, during their study on cherry angiomas in patients with neoplasia [1]. However, Hollander was the first to specifically associate SK with malignancy, although the eponym “Leser–Trélat” persists [1]. LST is typically characterized by the sudden onset of multiple SK in a symmetrical pattern on the back, sometimes associated with pruritus. The key clinical clue for identifying LTS is the suddenness of the eruption, which helps in differentiating LTS from the gradual development of SK that is common with aging. Histologically, SK as expression of LTS do not differ from SK in patients without malignancy [3]. LTS has been mostly associated with gastrointestinal adenocarcinoma [68], but also with several other malignancies, including pancreas, breast and lung carcinoma, MF, and cutaneous malignant melanoma (MM) [2,69,70,71]. LTS associated with MM has been reported in only five patients [72]. According to the literature, two cases were of unknown primary origin and three out of five patients had metastasis at the time of diagnosis [72]. Remarkably, the median Breslow thickness was 2.45 mm [72]. The sudden onset of multiple SK at the center or the periphery of a patch or a plaque in MF has also been reported [73]. However, in this case, an MF with unusual epidermal hyperplasia and high-risk human papillomavirus (HPV) should be ruled out [74,75]. LTS can be associated with other PD [54,76]. A pseudo LTS has been reported in patients without an underlying malignancy [77]. The pseudo LTS was firstly reported in 2004 and described as the exacerbation of pre-existing SK in a male patient with acute myelogenous leukemia on cytarabine [78]. Pseudo LTS has been later reported in association with COVID-19 [79], pityriasis rubra pilaris [80,81], lepromatous leprosy [82], and HIV [83]. However, the clinical significance of LTS is still controversial due to the frequent coexistence of SK and malignancy in older patients. Nevertheless, the sudden onset of SK, especially in patients under 30 years, is unusual and should be monitored. The pathophysiology of LTS is still unclear, but it is believed that cytokines and growth factors released by the neoplasm can stimulate the eruptive onset of SK. Indeed, the overexpression of EGF-α and TGF-α may play a role in the pathogenesis of LTS [3]. The treatment of the underlying malignancy leads to the resolution of LTS in about 50% of cases and symptomatic lesions can be treated by cryotherapy, curettage or shave excision [1].

### 3.3. Paraneoplastic Dermatomyositis

DM is a rare autoimmune disease, characterized by particular skin features and different degree of myopathy [84,85]. DM shows two peaks of incidence: the first one between 5 and 15 years and the other one 40 and 60 years [86]. DM is more frequent in the female population than in the male one [86]. The etiopathogenesis of DM is still unclear, but different factors, such as a genetic predisposition, environmental triggers, and immunological mechanisms, have been reported to play a role in the development of DM [87,88]. DM is characterized by T-cell-mediated myotoxicity and complement-mediated microangiopathy [87,88]. It has been thought that the primary targets in DM are the endomysial capillaries that are attacked by the membranolytic attack complex and C4b and C5b–9 fragments [87,88]. However, the specific target antigens and crucial triggers that lead to the development of DM are still unknown. According to the criteria proposed by Bohan and Peter (Table 4), particular skin manifestations are mandatory for the diagnosis of DM [89,90]. 

**Figure 3:**
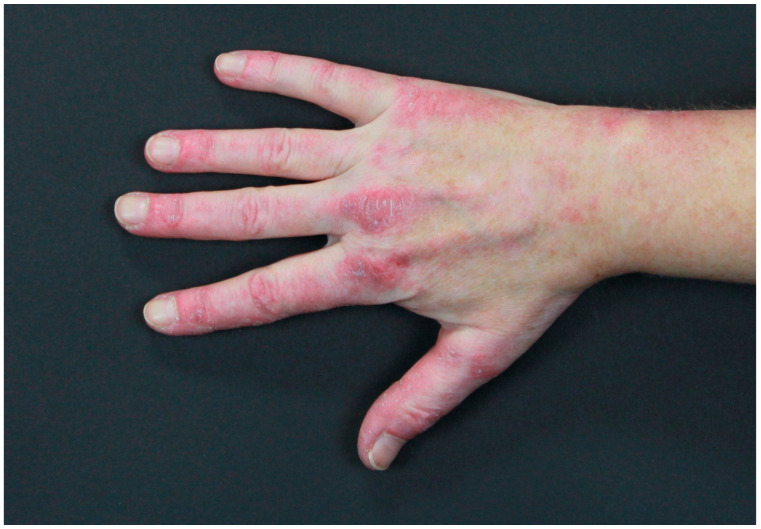
Gottron’s papules in a female patient with lung adenocarcinoma.

DM cutaneous features can be mainly classified into three groups, namely pathognomonic, characteristic, and compatible skin features [85,91]. Gottron’s papules (Figure 3) and Gottron’s sign belong to the pathognomonic skin features [85,91]. 

**Figure 4:**
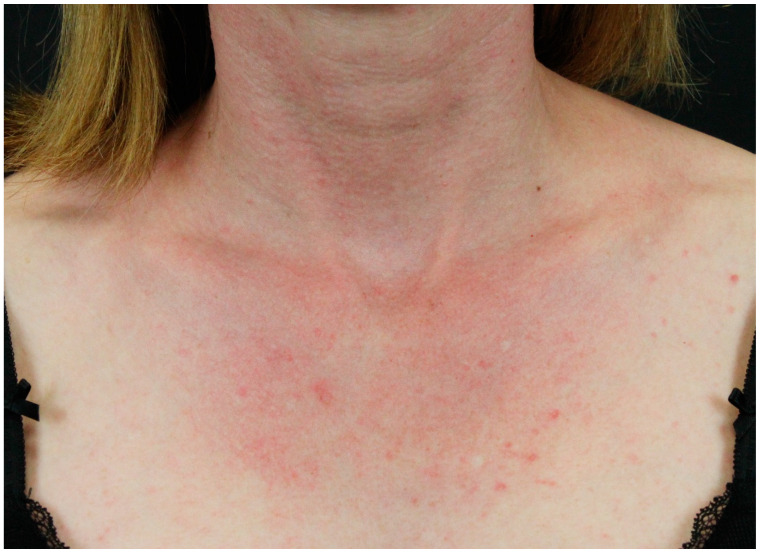
V-sign in a female patient with colorectal adenocarcinoma.

The first manifestations are slightly infiltrated, purplish, lesions on erythematous skin over bony prominences, mainly on the metacarpophalangeal, interphalangeal, and distal interphalangeal joints, while the last manifestation is represented by erythematous macules in a linear arrangement, mainly on the dorsal and lateral side of hands and fingers. Furthermore, facial heliotrope rash, shawl- and V-sign (Figure 4), and nail-fold changes (Keining’s sign), and scaly dermatitis of the scalp have been classified in the group of characteristic skin manifestations [85,91]. 

Facial heliotrope rash is a symmetric pruriginous purplish erythema with oedema that involves mainly the upper eyelids. The shawl- and V-signs show as a erythematous maculopapular rash of the upper back and deltoids and of the V area of upper chest, respectively. Periungual telangiectasia with dystrophic or overgrowth cuticles and small hemorrhagic infarcts are known as Keining’s sign. Lastly, poikiloderma (the association of atrophy, dyspigmentation, and telangiectasia), holster sign (erythema of hips and lateral thighs) and raccoon sign (symmetric periorbital dusk erythema associated with oedema) belong to the compatible skin features [85,91]. DM can be associated with underlying neoplasia. In a recent meta-analysis, an underlying neoplasm was detected in 40% of DM patients [92]. Lung and gastrointestinal malignancies have been mostly detected in Caucasian patients with paraneoplastic DM, while malignancy of nasopharynx is mostly detected in Asiatic patients [93,94]. Therefore, periodic cancer screening is mandatory in DM patients, but it is still under debate how frequent and which methods should be used for the screening [92]. In this regard, “blind screening” (investigations performed in the absence of target symptoms) was reported as useful by several authors [92,95]. Moreover, it has been reported that the risk of malignancy in DM patients is higher in the first year after diagnosis and especially in the first three months [96]. Noteworthy, accurate cancer screening should be performed in DM patients with anti-transcription intermediary factor 1 (TIF1)-γ autoantibodies, which are associated with a higher risk of underlying malignancies [97,98]. Autoantibodies are useful biomarkers to predict the prognosis and the systemic involvement in DM patients, but not all DM-associated autoantibodies are specific [84,85]. Indeed, myositis-specific antibodies (MSAs) are detected only in patients with idiopathic inflammatory myositis, while myositis-associated autoantibodies can be detected in both patients with myositis and other autoimmune diseases, such as systemic lupus erythematosus [84,85]. Furthermore, MSAs are not yet included in the diagnostic criteria of DM [85]. Systemic corticosteroids and steroid-sparing immunosuppressive drugs are considered the main therapy for DM [24,85]. In the therapeutic management of DM, several factors should be considered, such as the age of the patient, disease activity, and comorbidities. Indeed, a delay in starting proper therapy can lead to a worse prognosis and outcome [24,85]. Furthermore, a complete excision of the underlying neoplasm should be performed [24,85]. Because of its different skin features, several dermatoses should be ruled out according to the involved area, such as contact dermatitis, flagellate dermatitis, seborrheic dermatosis and systemic lupus erythematosus [85,99].

### 3.4. Pyoderma Gangrenosum

**Figure 5:**
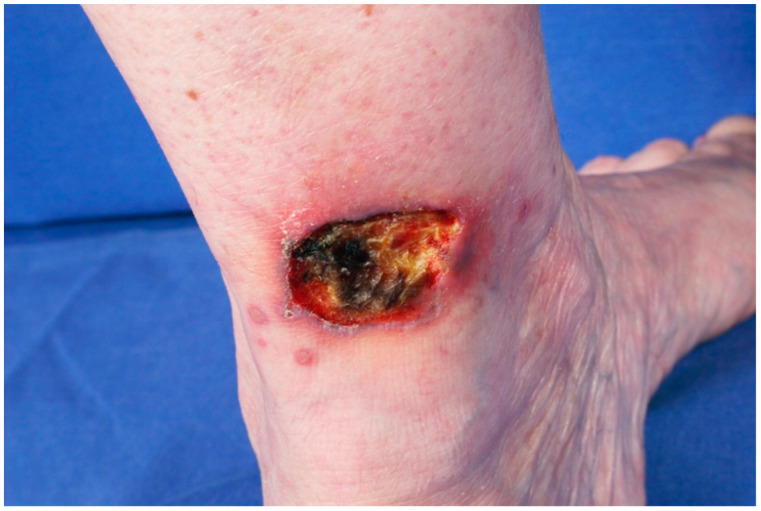
Ulcerative pyoderma gangrenosum in a male patient with myelodysplastic syndrome.

PG belongs to the group of neutrophilic disorders, and it is usually characterized by painful ulcerations with violaceous and undermined borders [100]. PG presents most frequently in elderly individuals [100], although about 4% of PG cases have been reported in the pediatric population [101]. PG more frequently affects the female population, with a sex ratio of 2:1 [100]. In a large cross-sectional analysis, an incidence of 5.8–20 PG per 100,000 inhabitants per year and a prevalence of 58 cases per 1,000,000 adults have been reported [102]. The clinical presentation of PG is variable. The most common form of PG is the ulcerative form, which is characterized by rapidly progressing painful ulceration with violaceous undermined borders (Figure 5) [103,104].

**Figure 6:**
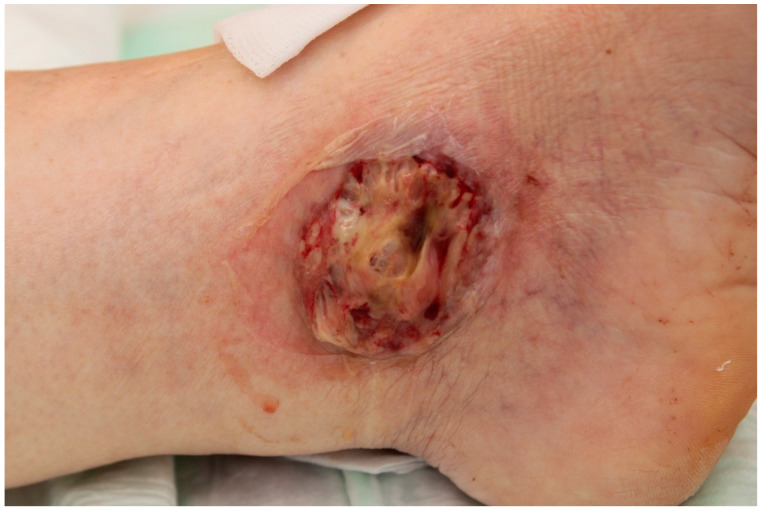
Bullous pyoderma gangrenosum in a male patient with acute myeloid leukaemia.

Bullous PG is characterized by blisters that quickly evolve into ulcers (Figure 6), while pustular PG is characterized by pustules that arise at the edges of an ulcer [103,104].

Finally, vegetative PG shows granulomatous or wart-like features [103,104]. Typically, PG involve the lower legs and can present after trauma or surgical procedures [103,104]. In most cases, PG is associated with underlying diseases, like inflammatory bowel diseases, arthritis and hematological disorders [103,104]. Indeed, in up to 10% of patients, PG arises in patient with hematological malignancies [105,106]. 

Nevertheless, PG can occur also in patients with solid malignancies, like ductal breast carcinoma [107]. Finally, PG can be a skin feature of several syndromes, like PAPA (pyogenic arthritis, pyoderma gangrenosum, acne) or PsAPASH (psoriatic arthritis, pyoderma gangrenosum, acne, hidradenitis suppurativa) syndrome [100]. The pathogenesis of PG is still largely unknown. Growing evidence suggests that patients with a genetic predisposition and abnormal activation of the innate immune system can develop PG [108]. Indeed, mutation of proline–serine–threonine phosphatase-interacting protein 1 gene (PSTPIP1) leads to an activation of the inflammasome and the uncontrolled production of interleukin (IL)-1β, which is related to the development of PAPA syndrome [109]. Furthermore, the overproduction of several inflammatory mediators, like IL-8, IL-17, IL-36, and TNF-α, plays an important role in PG pathogenesis [108,110,111]. In addition, the presence of clonal T-cell expansion has been reported in lesions of PG, which supports the role of aberrant T-cell response in the pathogenesis of PG [108,112]. In paraneoplastic PG, it has been supposed that the tumor-related inflammation can dysregulate the activity of neutrophils that leads to an excessive immune response [108]. Regarding the therapy, wound care is of pivotal importance to avoid the infection of the ulcer [108]. Topical therapies, like clobetasol propionate or calcineurin inhibitors, could be effective in mild cases [113]. However, in PG associated with malignancies or autoinflammatory syndrome, the main therapy depends on the associated disease [113]. PG should be differentiated from several other ulcerative diseases, including extraintestinal Morbus Crohn, several types of systemic vasculitis, and vascular ulceration of the lower legs [105].

### 3.5. Sweet’s Syndrome 

**Figure 7:**
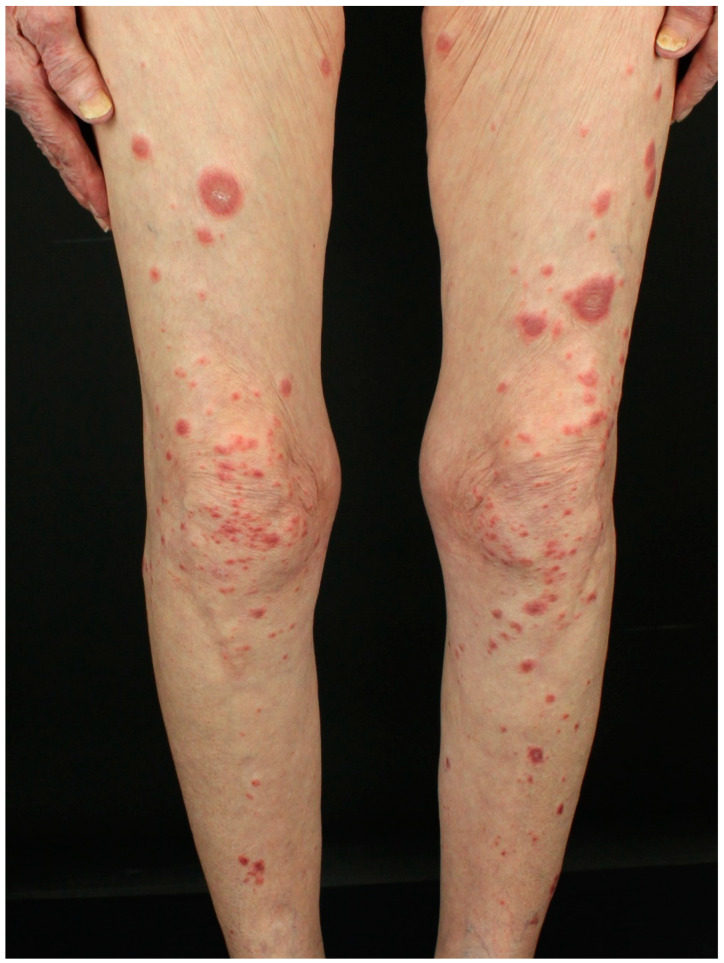
Infiltrated erythematous nodules involving the lower limbs in a female patient with acute myeloid leukemia.

Acute febrile neutrophilic dermatosis, also known as SS, was initially described in 1964 by Robert Douglas Sweet [114]. SS belongs to the group of neutrophilic dermatoses, which are haracterized by infiltration of neutrophils in the skin [115,116]. Clinically, SS shows erythematous tender papules or nodules, mainly involving the trunk and upper limbs (Figure 7), associated with fever, asymmetric polyarthralgia, and other uncommon systemic features, like myocarditis and meningitis [115,116].

SS can be idiopathic, or it can develop in association with other diseases, such as inflammatory bowel diseases, pyoderma gangrenosum and infections (toxoplasmosis, tuberculosis) [115,116]. Furthermore, SS can be induced by drugs, including antibiotics (minocycline, trimethoprim–sulfamethoxazole), antiepileptics (carbamazepine, diazepam), and immunosuppressants (azathioprine) [115,116]. In addition, SS can be associated with underlying neoplasms, mostly with hematological ones [1,2]. Paraneoplastic SS was first described by Cohen and Kurzrock and accounts for about 20% of SS cases [3,117,118]. Acute myeloid leukemia is the most frequently described neoplasia associated with paraneoplastic SS [119]. In paraneoplastic SS, an over-production and dysregulation of different cytokines and hormones have been reported, including IL-1, IL-3, IL-6, IL-8, granulocyte colony-stimulating factor (G-CSF), and granulocyte macrophage colony-stimulating factor (GM-CSF) [120]. Histologically, paraneoplastic SS cannot be differentiated from non-paraneoplastic SS and it is characterised by a neutrophilic infiltrate, composed to a lesser extent by macrophages and T lymphocytes, with leukocytoclasia, endothelial swelling, and focal erythrocyte extravasation [121]. The diagnosis of SS relies on the presence of both major (sudden onset of painful erythematous plaques or nodules and detection of a dense neutrophilic infiltrate without evidence of leukocytoclastic vasculitis) and two minor criteria, as reported in the table (Table 5) [116]. Paraneoplastic SS usually regresses after treating the underlying neoplasia [116]. However, in refractory cases, the first-line therapy is represented by systemic corticosteroids [116]. In addition, potassium iodide and colchicine have also been reported as alternative first-line therapies [116]. Differential diagnoses include psoriasis vulgaris, psoriasis guttata, and viral exanthema, among others [116]. In these cases, accurate detection of anamnesis can be extremely useful to make the correct diagnosis. 

## 4. Conclusions

Skin manifestations in patients with underlying neoplasia are extremely polymorphous. Their prompt recognition can lead to an earlier diagnosis of the underlying neoplasia and to an earlier start of the appropriate therapy. PAMS and paraneoplastic DM are two of the most paradigmatic PDs that should be promptly diagnosed to start adequate systemic therapy. Therefore, dermatologists should be able to recognise PD.

## Figures and Tables

**Table 1:** Criteria proposed by Curth to identify a paraneoplastic dermatosis [1,2,3].

Criterion
The onset of dermatosis must be related to the beginning of the neoplasia
Skin manifestations and neoplasia must show a parallel course
The dermatosis is not related to a genetic syndrome
A specific dermatosis occurs in patients with a specific tumor
The dermatosis is rare in the general population
High frequency of association between the dermatosis and the underlying neoplasia

**Table 2:** Clinical association between underlying malignancies and paraneoplastic dermatoses [1,2,3].

**Obligate Paraneoplastic Dermatosis**	**Neoplasia**	**Involved Molecular Factors**
Necrolytic migratory erythema	Glucagonoma and small cell lung cancer	Increased level of arachidonic acid Deficit of niacin
Paraneoplastic autoimmune multiorgan syndrome	Castleman’s disease, chronic lymphocytic leukemia, non-Hodgkin’s lymphoma, thymoma	
**Facultative Paraneoplastic Dermatosis**	**Neoplasia**	
Leser–Trélat’s Sign	Colorectal and gastric carcinoma	EGF-α, IGF-1, TGF-α
Malignant acanthosis nigricans	Gastrointestinal adenocarcinoma, hepatocellular carcinoma, pancreatic adenocarcinoma	EGF-α, FGF, IGF-1, MSH-α, TGF-α
Paraneoplastic dermatomyositis	Lung and ovarian adenocarcinoma	
Pyoderma gangrenosum	Leukemia, myelodysplastic syndrome, myeloma,	Fas, FasL, IL-1b, IL-8, IL-17, IL-23
Sweet’s syndrome	Acute myelogenous leukemia, myelodysplastic syndrome	G-CSF, GM-CSF, IL-1, IL-3, IL-6, IL-8
Tripe palms	Gastrointestinal and lung adenocarcinoma	EGF-α and TGF-α
**Other Paraneoplastic Dermatoses**	**Neoplasia**	
Acquired hypertrichosis lanuginosa	Breast, colorectal and lung adenocarcinoma	FGF
Acquired ichthyosis	Hodgkin’s lymphoma and lung adenocarcinoma	Impaired vitamin A metabolism TGF-α
Paraneoplastic acrokeratosis	Squamous cell carcinoma of several anatomic districts (e.g., esophagus, larynx, lung, tongue)	EGFR, IGF-1, TGF-α

Abbreviations: Epidermal growth factor alpha (EGF-α), epidermal growth factor receptor (EGFR), Fas ligand (FasL), fibroblast growth factor (FGF), granulocyte colony-stimulating factor (G-CSF), granulocyte macrophage colony-stimulating factor (GM-CSF), insulin growth factor-like (IGF-1), interleukin (IL), melanocyte-stimulating hormone (MSH) and tumour growth factor alpha (TGF-α).

**Table 3:** Diagnostic criteria according to S2k guidelines by European Academy of Dermatology and Venereology for Paraneoplastic Autoimmune Multiorgan Syndrome [43].

Clinical Criteria	Major Laboratory Criteria	Minor Laboratory Criteria
Underlying neoplasia	Detection of autoantibodies against desmoplakin, envoplakin, periplakin and/or A2ML1 by ELISA, IB or IP	Detection of linear or granular IgG and/or C3 along the BMZ by DIF
Chronic erosive mucositis	Detection of intercellular IgG deposition by IIF on rat bladder	Staining of the cytoplasmatic cell membrane of keratinocytes by IIF
Polymorphic skin lesions, including pemphigus-like and lichenoid lesions		Detection of anti-Dsg autoantibodies and at least one of the following autoantibodies (anti-desmocollin, anti-epiplakin, anti-plectin, anti-BP180 and anti-BP230) by ELISA, IB or IP

Legend: α-2 microglobulin-like 1 (A2ML1), basal membrane zone (BMZ), bullous pemphigoid (BP), desmoglein (Dsg), direct immunofluorescence (DIF), enzyme-linked immunosorbent assay (ELISA), immunoblotting (IB), immunoprecipitation (IP) and indirect immunofluorescence (IIF).

**Table 4:** Diagnostic criteria for dermatomyositis according to Bonham and Peter [89,90].

Criterion	Details
1. Symmetric proximal muscle weakness	With or without dysphagia and/or diaphragmatic weakness
2. Elevation of skeletal muscle enzyme levels	Creatine kinase (CK); aspartate transaminase (AST); alanine transaminase (ALT); lactate dehydrogenase (LDH)
3. Abnormal electromyography results	Polyphasic motor unit action potentials (MUAPs); fibrillation potentials; positive sharp waves; increased insertional irritability; repetitive high-frequency discharges
4. Muscle biopsy abnormalities	Histopathologic findings of muscle degeneration/regeneration/necrosis; interstitial mononuclear infiltrates
5. Typical skin rash of dermatomyositis	Heliotrope rash or Gottron’s papules

Definite dermatomyositis: criterion nr. 5 and at least 3 of criteria nr. 1–4; probable dermatomyositis: criterion nr. 5 and at least 2 of criteria nr. 1–4; possible dermatomyositis: criterion nr. 5 and at least 1 of criterion nr. 1–4.

**Table 5:** Diagnostic criteria for Sweet’s syndrome [116].

Major Criteria *
Abrupt onset of painful erythematous plaques or nodules
Histopathologic evidence of a dense neutrophilic infiltrate without evidence of leukocytoclastic vasculitis
**Minor Criteria**
Fever
Association with: Underlying hematological or visceral malignancy Inflammatory bowel disease Pregnancy Infection of the respiratory or gastrointestinal tract Vaccination
Excellent clinical response to systemic corticosteroids or potassium iodide
Elevation of three of the four laboratory values: Erythrocyte sedimentation rate C-reactive protein Absolute leukocytosis Peripheric neutrophilia

* Both the major criteria and two of the four minor criteria must be present for the diagnosis.

## Abbreviations

The following abbreviations are used in this manuscript:

A2ML1	α-2 microglobulin-like 1
AN	Acanthosis nigricans
BMZ	Basal membrane zone
BO	Bronchiolitis obliterans
BP	Bullous pemphigoid
DIF	Direct immunofluorescence
Dsg	Desmoglein
DM	Dermatomyositis
EGF-α	Epidermal growth factor α
ELISA	Enzyme-linked immunosorbent assay
EGFR	Epidermal growth factor receptor
EGF	Epidermal growth factor
ERK	Extracellular-signal-regulated kinase
FGF	Fibroblast growth factor
FGFR3	Fibroblast growth factor receptor 3
G-CSF	Granulocyte colony stimulating factor
GM-CSF	Granulocyte macrophage colony stimulating factor
HOA	Hypertrophic osteoarthropathy
HPV	Human papillomavirus
IB	Immunoblotting
IBD	Inflammatory bowel diseases
IIF	Indirect immunofluorescence
IL	Interleukin
IGF-1	Insulin growth factor 1
IP	Immunoprecipitation
LTS	Leser–Trélat’s sign
MAN	Malignant acanthosis nigricans
MAPK	Mitogen-activated protein kinase
MSH	Melanocyte-stimulating hormone
MF	Mycosis fungoides
MM	Malignant melanoma
MSA	Myositis-specific antibodies
NME	Necrolytic migratory erythema
PAMS	Paraneoplastic autoimmune multiorgan syndrome
PAPA	Pyogenic arthritis, pyoderma gangrenosum, acne
PsAPASH	Psoriatic arthritis, pyoderma gangrenosum, acne, hidradenitis suppurativa
PD	Paraneoplastic dermatosis
PF	Pemphigus foliaceus
PG	Pyoderma gangrenosum
PNP	Paraneoplastic pemphigus
PV	Pemphigus vulgaris
PSTPIP1	Proline–serine–threonine phosphatase-interacting protein 1 gene
SK	Seborrheic keratoses
SS	Sweet’s syndrome
TGF-α	Tumor growth factor α
TIF1-γ	Transcription intermediary factor 1 γ
TNF-α	Tumor necrosis factor α
TP	Tripe palms
